# Bloodstream infections in patients undergoing extracorporeal membrane oxygenation

**DOI:** 10.12669/pjms.36.6.2882

**Published:** 2020

**Authors:** Jian-rong Wang, Jin-yu Huang, Wei Hu, Xue-ying Cai, Wei-hang Hu, Ying Zhu

**Affiliations:** 1 Jian-rong Wang, MS. Department of Critical Care Medicine, The Affiliated Hangzhou Hospital of Nanjing Medical University, Zhejiang, China; 2 Jin-yu Huang, MD. Department of Cardiology, The Affiliated Hangzhou Hospital of Nanjing Medical University, Zhejiang, China; 3 Wei Hu, MS. Department of Critical Care Medicine, The Affiliated Hangzhou Hospital of Nanjing Medical University, Zhejiang, China; 4 Xue-ying Cai, MS. Department of Critical Care Medicine, The Affiliated Hangzhou Hospital of Nanjing Medical University, Zhejiang, China; 5 Wei-hang Hu, MS. Department of Critical Care Medicine, Zhejiang Hospital, Zhejiang, China; 6 Ying Zhu, MS. Department of Critical Care Medicine, The Affiliated Hangzhou Hospital of Nanjing Medical University, Zhejiang, China

**Keywords:** Bloodstream infection, Extracorporeal membrane oxygenation, Pathogen, Risk factors, Prognosis

## Abstract

**Objective:**

We aimed to evaluate the incidence, risk factors, and prognosis of bloodstream infections (BSIs) during extracorporeal membrane oxygenation (ECMO) treatment in a Chinese population.

**Methods:**

Patients receiving ECMO treatment from January 2013 to August 2019 were retrospectively studied. The incidence of BSIs was calculated. The clinical characteristics between patients with a BSI (BSI group) and without a BSI (non-BSI group)

**Results:**

Among 69 included patients, 19 (27.5%) developed at least one BSI. Gram-negative bacteria (73.7%) were mainly responsible for the BSIs, with *Klebsiella pneumoniae* (6/19, 31.5%) ranking as the top related pathogen. The BSI group had a greater proportion of methicillin-resistant *Staphylococcus aureus* (MRSA) prophylactic regimens (52.6% vs. 26.0%, P = 0.036), a higher pre-ECMO Sequential Organ Failure Assessment (SOFA) score (11 vs. 8, P = 0.008), more applications of continuous renal replacement therapy (CRRT) during ECMO (63.1% vs. 36.1%, P = 0.042). Longer ECMO support duration, period of ventilator use before ECMO weaning and hospital stay were observed in the BSI group. The SOFA score (OR: 1.174; 95% CI: 1.039–1.326; P = 0.010) was an independent risk factor for BSIs.

**Conclusion:**

BSIs during ECMO therapy frequently involve Gram-negative bacteria. Stringent care and monitoring should be provided for patients with high SOFA scores.

## INTRODUCTION

Extracorporeal membrane oxygenation (ECMO) is one of the most important strategies to treat severe acute respiratory failure or cardiac failure. The application of ECMO in adults has increased rapidly since the influenza A H1N1 epidemic and the completion of the CESAR trial.^1^

Despite the growing implementation of adult ECMO, mortality due to severe acute respiratory failure or cardiac failure remains relatively high. The overall survival rate for these patients in the extracorporeal life support organization (ELSO) registry was 56%, and it varied depending on the patient population and health care providers.^2^ Nosocomial infection is a complication commonly seen and usually contribute to a high mortality rate. Multiple factors increase the risk of nosocomial infection in patients receiving ECMO.^3^ Furthermore, the incidence of bloodstream infections (BSIs) remains substantial, thus impacting the prognosis of patients treated with ECMO.^4^,^5^ Therefore, the management of patients with a BSI during ECMO remains a challenge.

A thorough understanding of the clinical features of BSIs may improve the prognosis of patients receiving ECMO.^6^ Therefore, in this study, we aimed to explore the incidence, risk factors, and prognosis of Chinese patients undergoing ECMO with BSIs.

## METHODS

**Fig.1:**
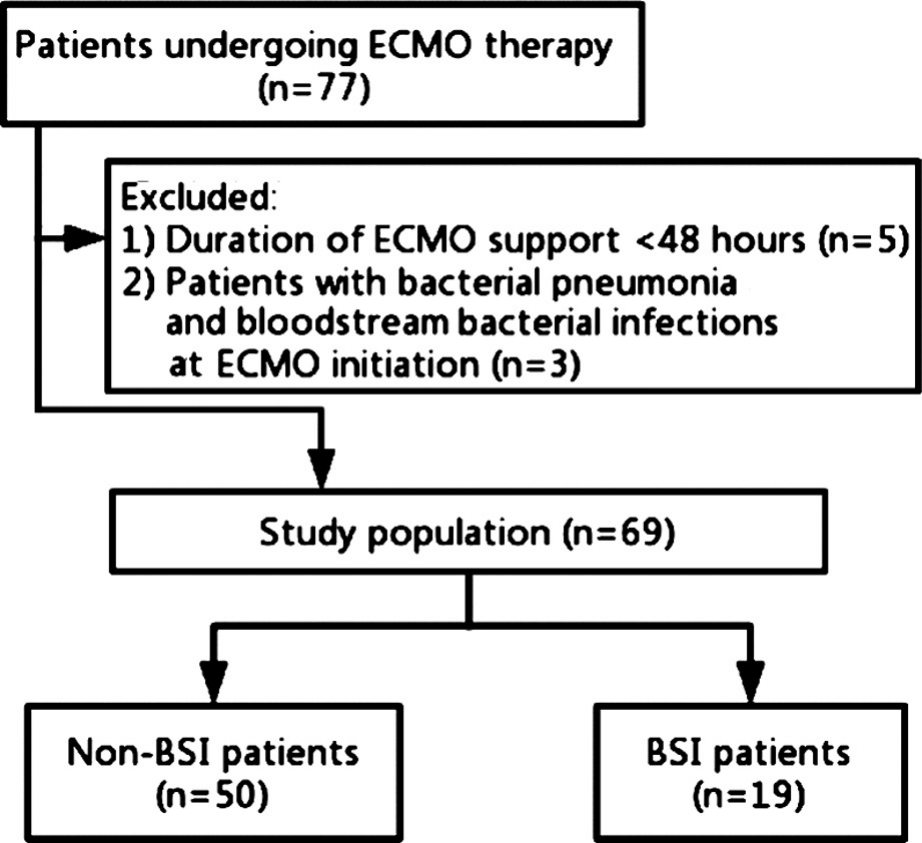
The study flowchart.

All adult patients (n = 77) requiring ECMO at the Department of Critical Care Medicine, Affiliated Hangzhou Hospital of Nanjing Medical University, from January 2013 to August 2019 were retrospectively reviewed. Finally, a total of 69 patients receiving veno-venous or veno-arterial ECMO owing to cardiopulmonary failure were included in this study (Fig.1). Demographic, clinical, and prognostic parameters were collected.

This study was approved by the local Hospital Ethics Committee (No. 2019-007-01). The informed consent was waived due to the retrospective nature of this study.

### Definitions and criteria

The survival-to-discharge rate was defined as the primary outcome. ECMO-related nosocomial pneumonia was defined as pneumonia occurring in patients receiving ECMO for more than 48 hours or withdrawn within 48 hours.^7^ We classified the prophylactic antibiotic regimens into two categories (Table-I).

**Table I:** Regimens of prophylactic treatment with antibiotics.

Group	Prophylactic Antibiotics	No. of patients (n = 69)
Group 1	Piperacillin/tazobactam+teicoplanin	6
	Piperacillin/tazobactam+linezolid	6
	Piperacillin/tazobactam+daptomycin	6
	Piperacillin/tazobactam+vancomycin	4
	Imipenem/cilastatin+teicoplanin	1
Group 2	Piperacillin/tazobactam	32
	Cefoperazone/sulbactam	2
	Meropenem	2
	Imipenem/cilastatin	2
	Piperacillin/tazobactam+moxifloxacin	2
	Cefoperazone/sulbactam+moxifloxacin	1
	Cefmetazole	1
	Cefuroxime	1
	Amoxicillin/clavulanic acid	1
	Moxifloxacin	1
	Cefatriaxone	1

### Statistical analysis

Categorical variables were expressed as frequencies and percentages, and continuous variables were expressed as the median (range) or mean ± standard deviation, as appropriate. Categorical variables were compared between groups using the χ^2^ or Fisher’s exact test, and continuous variables were compared using the Mann–Whitney U test. Multivariate logistic regression analyses were conducted to determine the independent predictive factors of nosocomial pneumonia. The variables with P< 0.1 in the univariate analysis were included in the multivariate analysis with a forward stepwise model. All statistical analyses were performed using SPSS 21.0 (IBM Corp., Armonk, NY, USA). All tests were two-tailed, and a value of P<0.05 was considered statistically significant.

## RESULTS

The baseline characteristic was displayed in Table-II. Myocarditis was the most common reason for ECMO treatment. A total of 30 (42.5%) patients with ECMO support developed 43 episodes of nosocomial infection, with an incidence of 76.0 infections per 1000 days of ECMO. Specifically, BSIs were observed in 19 patients (27.5%) with an incidence of 33.6 infections per 1000 days of ECMO. In addition, 14 (20.3%) patients experienced nosocomial pneumonia, and 10 (14.5%) patients had a urinary tract infection.

**Table-II:** Baseline characteristics.

Characteristic	Study population (n = 69)
***Demographic data***	
Age, years	42 (18–77)
Gender, female	26 (37.7)
***Primary disease***	
Myocarditis	40 (58.0%)
Coronary heart disease	13 (18.8%)
Pneumonia (viral/interstitial/aspergillus)	6 (8.7%)/1 (1.4%)/1 (1.4%)
Pulmonary contusion	3 (4.3%)
Pulmonary arterial hypertension	2 (2.9%)
Lung cancer with airway obstruction	1 (1.4%)
Aortic dissection	1 (1.4%)
Allergic shock	1 (1.4%)
MRSA prophylactic regimens	23 (33.3%)
***Laboratory findings (Pre-ECMO)***	
White blood cell, 10^3^/mm^3^	11.5 (2.2–37.7)
Hemoglobin, g/dL	120.0 (61–176)
Platelets, 10^3^/mm^3^	174 (11–857)
CRP, mg/dL	32 (1–194)
Lactate, mM	3.8 (1.0–20.0)
Total bilirubin, mg/dL	14.6 (4.3–131)
Creatinine, mg/dL	98.5 (44–466)
Pre-ECMO SOFA score	8 (0–22)
Pre-ECMO ventilator support, days	0 (0–9)
Pre-ECMO ICU stay, days	0 (0–4)
Pre-ECMO hospital stay, days	0 (0–14)
Veno-arterial mode	58 (84.1%)
Ventilator duration before ECMO weaning, days	7 (0–32)
ECMO support duration, h	154 (55–727)

Gram-negative bacteria (73.7%) were mainly responsible for the BSIs, with *Klebsiella pneumoniae* (6/19, 31.5%) ranking as the top related pathogen (Table-III). The BSI group had greater proportions of MRSA prophylactic regimens [10 (52.6%) vs. 13 (26.0%); P = 0.036] and CRRT [12 (63.1%) vs. 18 (36.0%); P = 0.042] than the non-BSI group. The ECMO support duration [(8.0 (range: 3.6–26.0) vs. 5.8 (range: 2.3–30.3) days; P = 0.001] and ventilator duration before ECMO weaning [10 (range: 3–26) vs. 6 (range: 0–32) days; P = 0.001] in the BSI group were longer than those in the non-BSI group. Similarly, a longer length of hospitalization was observed in the BSI group than in the non-BSI group [25 (range: 10–39) vs.17.5 (range: 2–42) days; P = 0.029] (Table-IV).

**Table-III:** Pathogens of BSIs during ECMO support.

Microorganism species	BSI (n = 19)
***Gram-negative pathogens***	
*Klebsiella pneumonia*	6
*Enterobacter aerogenes*	2
*A. baumannii*	1
*Burkholderia cepacia*	1
*Pseudomonas aeruginosa*	1
*Enterobacter cloacae*	1
*Serratia marcescens*	1
*Stenotrophomonas maltophilia*	1
***Gram-positive pathogens***	
*Staphylococcus epidermidis*	2
*Enterococcus faecium*	1
***Fungi***	
*Candida albicans*	1
*Candida parapsilosi*	1

**Table-IV:** Differences of characteristics between patients with and without BSIs during ECMO.

Characteristics	Without BSI (n = 50)	With BSI (n = 19)	P value
***Demographic characteristics***			
Age, years	40 (18–77)	44 (21–67)	0.872
Female	19 (38.0)	7 (36.8)	0.929
Smoking history	12 (24.0)	6 (31.6)	0.522
***Primary disease***			
Myocarditis	24 (48.0)	8 (42.1)	0.661
Respiratory failure	8 (16.0)	4 (21.1)	0.725
Coronary artery disease	13 (26.0)	6 (31.6)	0.643
***Laboratory findings (Pre-ECMO)***			
White blood cell, 10^3^/mm^3^	10.65 (2.20–36.7)	13.3 (5.2–37.7)	0.256
Hemoglobin, g/L	119.5 (61–176)	122 (70–174)	0.984
Platelets, 10^3^/mm^3^	175.0 (33.0–396.0)	140 (11–857)	0.100
Lactate, mM	3.8 (1.0–20.0)	2.3 (1–19)	0.859
Total bilirubin, μM	14.0 (4.3–131.0)	20.1 (7.9–127)	0.079
Creatinine, μM	94.0 (44–466)	124 (55–297)	0.070
CRP, mg/dL	22.5 (1–176)	59 (1–194)	0.192
***Pre-ECMO data***			
Pre-ECMO CPR	16 (32.0)	9 (47.5)	0.235
Veno-venous ECMO mode	8 (16.0)	4 (21.1)	0.725
Pre-ECMO SOFA score	8 (0–21)	11 (1-22)	0.008
Pre-ECMO ICU stay ≥ 1 day	12 (24.0)	6 (31.6)	0.522
Pre-ECMO ventilator duration ≥ 1 day	12 (24.0)	8 (42.1)	0.139
Pre-ECMO hospital stay ≥ 1 day	22 (44.0)	8 (42.1)	0.887
Pre-ECMO GCS	15 (3.0–15.0)	15 (3–15)	0.816
MRSA prophylactic regimen	13 (26.0)	10 (52.6)	0.036
***During ECMO data***			
IABP during ECMO	13 (26.0)	8 (42.1)	0.194
CRRT during ECMO	18 (36.0)	12 (63.1)	0.042
Corticosteroid (methylprednisolone) before and during ECMO, mg	545 (0–3100)	560 (0–2860)	0.793
ECMO support duration, days	5.8 (2.3–30.3)	8.0 (3.6–26.0)	0.001
***Before ECMO weaning data***			
ventilator duration before ECMO weaning, days	6 (0–32)	10 (3–26)	0.001
***Prognosis***			
ECMO weaning (Success)	40 (80.0)	8 (42.1)	0.147
hospital stay, days	17.5 (2–42)	25 (10–39)	0.029
Survival-to-discharge rate	33 (66.0)	8 (42.1)	0.532

The pre-ECMO SOFA score (OR: 1.174; 95% CI: 1.039–1.326; P = 0.010), was the independent risk factors for BSIs. Patients with a higher pre-ECMO SOFA score were more likely to experience a BSI (Table V & VI).

**Table-V:** Univariate analysis of risk factors for BSIs.

Risk factors	OR (95%CI)	P value
***Demographic characteristics***		
Age, years	1.000 (0.970–1.032)	0.980
Female gender	1.051 (0.352–3.135)	0.929
Smoker	1.462 (0.456–4.685)	0.523
***Primary disease***		
Myocarditis	0.788 (0.271–2.289)	0.661
Respiratory failure	1.400 (0.368–5.332)	0.622
Coronary artery disease	1.314 (0.414–4.171)	0.644
***Laboratory findings (Pre-ECMO)***		
White blood cell (10^3^/mm^3^)	1.033 (0.963–1.108)	0.366
Hemoglobin, g/L	0.998 (0.978–1.019)	0.880
Platelets (10^3^/mm^3^)	0.999 (0.994–1.004)	0.708
Lactate, mM	1.038 (0.931–1.158)	0.501
Total bilirubin, μM	1.017 (0.994–1.041)	0.152
Creatinine, μM	1.006 (0.998–1.014)	0.117
CRP mg/dL	1.007 (0.998–1.016)	0.122
***Pre-ECMO data***		
Pre-ECMO CPR	1.912 (0.650–5.626)	0.239
Veno-venous ECMO mode	1.400 (0.368–5.332)	0.622
Pre-ECMO SOFA score	1.174 (1.039–1.326)	0.010
Pre-ECMO ICU stay ≥1 day	1.462 (0.456–4.685)	0.523
Pre-ECMO ventilator duration ≥1 day	2.303 (0.753–7.047)	0.144
Pre-ECMO hospital stay ≥1 day	0.926 (0.318–2.694)	0.887
Pre-ECMO GCS	0.982 (0.886–1.088)	0.727
MRSA prophylactic regimen	3.162 (1.053–9.502)	0.040
***During -ECMO data***		
IABP during ECMO	2.070 (0.683–6.271)	0.198
CRRT during ECMO	3.048 (1.018–9.124)	0.046
Corticosteroid before and during ECMO, mg	1.000 (0.999–1.001)	0.877
ECMO support duration, days	1.103 (1.003–1.212)	0.043
***Before ECMO weaning data***		
ventilator duration before ECMO weaning, days	1.109 (1.021–1.206)	0.014
***Prognosis***		
ECMO weaning (Success)	0.429 (0.134–1.369)	0.153
hospital stay, days	1.065 (1.005–1.129)	0.034
Survival-to-discharge rate	0.708 (0.240–2.091)	0.532

**Table-VI:** Multivariate analysis of risk factors for BSIs.

Risk factors	OR (95%CI)	P value
Pre-ECMO SOFA score	1.174 (1.039–1.326)	0.010
MRSA prophylactic regimen		0.104
CRRT during ECMO		0.365
ECMO support duration, days		0.146
Ventilator duration before ECMO weaning, days		0.041

## DISCUSSION

The current retrospective study found that 27.5% of the patients experienced a BSI with Gram-negative bacteria as the predominant pathogen. Patients with a higher SOFA score were more likely to have a BSI. Prophylactic antibiotics with anti-MRSA activity may increase BSIs. Although BSIs were associated with a longer hospital stay, there was no significant correlation between BSI and mortality.

The BSIs prevalence in patients undergoing ECMO is reported to vary from 3% to 18%, based on the region, race, and disease status; the corresponding incidence ranges from 2.98 to 20.55 episodes per 1000 days of ECMO in adults.^8^,^9^ In the current study, 19 patients (27.5%) developed a BSI, with an incidence of 33.6 infections per 1000 days of ECMO, which seems to be higher than the incidence reported in previous studies. We speculate that an emergent catherization in an overcrowded and contaminated ER may be responsible for this higher BSIs rate.

In this study, BSIs were mostly caused by Gram-negative bacteria. We found that Gram-negative bacteria were responsible for 73.7% of the BSIs identified, with the leading pathogens being *K. pneumoni*a, *Enterobacteraerogenes*, and *Staphylococcusepidermidis*. Our results were partly consistent with recent studies from other single centers, which demonstrated that *Enterobacteriaceae* and *Acinetobacter baumannii* were the most common pathogens.^3^,^9^,^10^ In contrast, early data (1998–2008) from the ELSO Registry show that the main pathogens of nosocomial infection include the Gram-positive bacteria coagulase-negative *Staphylococci* (15.9%), *Staphylococcus aureus* (9.4%), *Candida* (12.7%), and *Pseudomonas aeruginosa* (10.5%).^1^ However, the specific discrimination criteria of infection from contamination in the ELSO registry as well as the site of infectious episodes were not reported, which may diminish the reliability of pathogens reported in the past decades.^7^ The shift towards more Gram-negative infections is thought to be related to the changing resistance patterns, biofilm formation, and exposure to multi-drug resistant bacteria in healthcare environments.^11^

Controversies still exist concerning the necessity of prophylaxis for ECMO treatment. In our research, all patients were given intravenous prophylactic antibiotics during the process of ECMO support. However, the incidence of overall infection was still high. Additionally, BSIs were more commonly seen in patients with anti-MRSA regimens. This increased likelihood of BSI in the anti-MRSA group is probably linked to dysbiosis (imbalance of guts microbial environment) of the intestinal flora and bacterial translocation. Therefore, we speculate that prescription of anti-MRSA medications to prevent BSIs should be used with caution in ECMO patients. Other studies also have shown that antibiotic prophylaxis did not reduce ECMO-related infection.^5^ Thus, the ELSO Infectious Disease Task Force does not recommend the administration of antimicrobial agents to prevent infectious complications during ECMO support and advises not to prolong it beyond 48 hours after cannulation.^12^

There is a higher risk of nosocomial infection with a longer ECMO duration. Several previous studies have reported a similar clear association between the risk of nosocomial infection and ECMO duration.^8^,^13^,^14^ Burket *et al*. have reported that the incidence of BSIs increased from 9.5 to 64.5 infections per 1000 days of ECMO for patients with ECMO lasting from 3–10 days to 21–30 days.^5^ Moreover, the duration of ventilator support before ECMO weaning was similarly related to BSI occurrence as long-term ventilator support increased the incidence of ventilator-associated pneumonia and secondary BSIs.^4^ Of note, BSIs may also prolong the duration of ECMO or the duration of ventilator use. In the currents study, patients with BSI had a significantly longer duration on ECMO (8 vs. 5.8 days) and ventilator duration before ECMO weaning (10 vs. 6 days) although we failed to prove that the duration of ECMO or ventilator duration was an independent risk factor for BSIs due to the limited sample size.

The pre-ECMO SOFA score, which reflects multiple organ function, was an independent risk factor for BSIs in the current study. This result confirmed the previous findings that a higher SOFA score before cannulation is an independent risk factor for overall infectious complications and for BSIs.^14^-^16^ Previous studies have also found an association between renal failure and BSIs in patients with veno-venous ECMO as well as a relationship between hepatic failure and BSIs in patients with veno-arterial ECMO.^17^ There are several explanations for these results. First, invasive procedures, medication, electrolyte disorders, and blood infusion may increase the risk of bacterial contamination. Second, organ failure may also lead to prolonged ECMO and ventilator support, which increase the possibility of infection. Therefore, ECMO support should be initiated as early as possible to restrain the development of multiple organ dysfunction.

The impact of BSIs on clinical outcomes is still largely unknown. Some studies have demonstrated a significantly elevated mortality in pediatric patients with BSIs compared with those without BSIs,^18^ but other studies of patients receiving veno-venous ECMO have shown that BSIs have no effect on mortality.^19^ In this study, the differences between the two groups were not significant, whereas the survival-to-discharge rates were 66% for the non-BSI group and 44.5% for the BSI group. Advances in sepsis care and earlier initiation of antibiotics may reduce the impact of BSIs on the patient prognosis.

### Limitations of the study

This study has several limitations that must be acknowledged. First, it was a retrospective, single center study, and the sample size was small. Second, the source of BSI was difficult to identify, and the ratio of primary BSI to secondary BSI was not reported. Third, our analysis only included the first episode of BSI and thus may underestimate the overall incidence of BSIs.

## CONCLUSION

Gram-negative bacteria are the predominant pathogens causing BSI during ECMO treatment. Severe organ failure increases the risk of BSI in patients receiving ECMO.

### Authors’ Contributions:

***Jian-rong Wang*** data analysis and wrote the manuscript.

***Jin-yu Huang*** contributed to interpretation of the data.

***Wei Hu*** designed most of the investigation.

***Xue-ying Cai, Wei-hang Hu, and Ying Zhu*** contributed to data collection and literature search.

All authors are accountable for the accuracy or integrity of the work.
